# Demographic Models and Behavioral Assessments Uncover Distinct Species Histories in the Pseudocryptic Nudibranch Genus *Hermissenda*

**DOI:** 10.1002/ece3.73045

**Published:** 2026-02-03

**Authors:** Miranda T. Dennis, Austin L. Ka’ala Estores‐Pacheco, Keilan Williams, Russell C. Wyeth, Ángel Valdés, Arne Ø. Mooers, Michael W. Hart

**Affiliations:** ^1^ Department of Biological Sciences Simon Fraser University Burnaby British Columbia Canada; ^2^ Department of Biological Sciences California State Polytechnic University Pomona California USA; ^3^ Department of Biology St. Francis Xavier University Antigonish Nova Scotia Canada

**Keywords:** demography, ecology, Heterobranch, niche overlap, prezygotic isolating barrier, speciation

## Abstract

The Pacific nudibranch *Hermissenda crassicornis* (sensu lato) is a well‐known model organism in neuroscience. This species was recently split into three pseudocryptic species based on differences in genetics, morphology, and behavior. We used ddRADSeq data from 33 individuals (2354 loci) and coalescent isolation‐with‐migration models alongside forward simulations to estimate and evaluate the demographic history of the clade. We inferred (1) a novel phylogenetic tree topology with the North American species as sisters, (2) relatively old divergence times (0.55 and 1.29 mya), (3) a much larger population size in the southern species *H. opalescens* , and (4) no recent or current gene flow between the sympatric species *H. crassicornis* and *H. opalescens* . Then we examined behavioral differences between sympatric *H. crassicornis* and *H. opalescens* to characterize possible mechanisms promoting their reproductive isolation. Mating experiments showed that both *H. crassicornis* and *H. opalescens* display assortative mating, consistent with the existence of a prezygotic reproductive barrier. Our results support the splitting of *H. crassicornis* (sensu lato) and reinforce the need to reassess previous studies that used the species complex as a model organism.

## Graphical abstract

We reconstructed the speciation history of the recently split pseudocryptic nudibranch genus *Hermissenda*, finding divergence times in the Pleistocene (1.29–0.55 mya). Demographic modeling and behavioral experiments show no gene flow and assortative mating between sympatric species *H. crassicornis* (s.s.) and *H. opalescens* , consistent with prezygotic isolation, and show large differences in effective population size. Our results support the splitting of *H. crassicornis* (s.l.) and reinforce the need to reassess previous studies that used the species complex as a model organism.

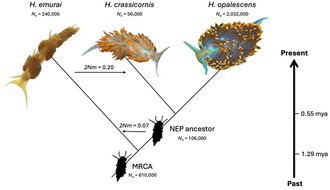

## 1 Introduction

Identifying and characterizing pseudocryptic species is of considerable importance for ecological and evolutionary analyses (Luttikhuizen and Dekker 2009; Fišer et al. 2018). Pseudocryptic species are defined as those which are distinguished morphologically only following analyses of molecular data (Sáez et al. 2003; Lindsay and Valdés 2016). Such species complexes imply slow rates of divergence in some traits that make the species difficult to distinguish by phenotype (if the species diverged long ago), or rapid divergence of other traits that lead to reproductive isolation (and speciation if the species diverged recently). The analysis of genetic data to characterize divergence times, effective population sizes, and gene flow among pseudocryptic species may be one of the most effective ways to begin to explore these islands of biodiversity by identifying abiotic or biotic factors associated with the timing of speciation or the magnitude of differences in the size or reproductive isolation among species (Mann and Evans 2008).

Several pseudocryptic species complexes have recently been found among Pacific Nudibranchia (Mollusca: Gastropoda: Heterobranchia). The dorid nudibranch *Diaulula sandiegensis* (sensu lato) was found to be a pseudocryptic species pair, *D. sandiegensis* (sensu stricto) and *D. odonoghuei*, that may have diverged 1.7–1.5 mya in the early Pleistocene, coinciding with ice sheet expansion approximately 1.5 mya (Lindsay et al. 2016). Similarly, the North Pacific clown nudibranch, *Triopha catalinae* (sensu lato), includes two subtly distinct morphotypes including one ranging from South Korea to Southern California ( *T. modesta* ) and a second species that is restricted to the eastern Pacific from Southeast Alaska to Baja California ( *T. catalinae* sensu stricto; Jung et al. 2020). Like the pseudocryptic species of *Diaulula*, these patterns are consistent with the hypothesis that Pleistocene glacial cycles may play a role in these species divergences (Jung et al. 2020).

These recent discoveries are surprising: nudibranchs have a high profile in both popular and scientific culture in part because of their bright distinctive colouration and anthropomorphic esthetic, and in part due to their numerous distinctive adaptations and highly derived phenotypes compared to their relatives among the shelled gastropods (Dean and Prinsep 2017). Such charismatic and frequently collected organisms are not expected to harbor unrecognized species diversity. Characterizing the demographic history of divergence among such cryptic species might therefore lead to insights into the (slow) rate of divergence of many of their phenotypic traits or the (rapid) evolution of reproductive isolation between them.

Studying unrecognized species diversity is particularly important in some nudibranchs that are also used as model organisms in neurobiological studies (Getting and Willows 1974; Crow and Alkon 1978). The aeolid nudibranch *Hermissenda crassicornis* (eschscholtz, 1831), in the recently revised family Myrrhinidae (Martynov et al. 2019), has been used widely in studies of classical conditioning in response to light and vestibular stimuli (Dennis 1967; Crow and Alkon 1978; Tamvacakis et al. 2015) and in studies of associative learning, memory, neural circuit structure, and sensory and motor neuron physiology (Blackwell 2006; Tamvacakis et al. 2015; Lindsay and Valdés 2016).

In 2016, *H. crassicornis* (sensu lato) was discovered to be a pseudocryptic species complex consisting of the genetically distinct but morphologically similar species *H. crassicornis* (eschscholtz, 1831; sensu stricto) and *H. opalescens* (cooper, 1863) in the northeastern Pacific, and *H. emurai* (baba, 1937; Lindsay and Valdés 2016) in the northwestern Pacific. This unappreciated diversity could lead to complexities in interpreting the substantial collection of neurobiological studies that have assumed a single species over the last 50 years that included individual nudibranchs collected from diverse localities in the Northeastern Pacific Ocean, including localities inside of *H. crassicornis* and *H. opalescens* ranges.

Lindsay and Valdés (2016) suggested that the northern species *H. crassicornis* and the southern species *H. opalescens* have a narrow range overlap in California from Point Reyes to Bodega Bay. However, Merlo et al. (2018) documented a much broader range for *H. opalescens* , including southern British Columbia, Canada, and suggested that this represented a recent range extension during an El Niño year and a dramatic increase in the range overlap of these two species. To complicate matters further, more recent surveys have shown *H. crassicornis* occurrences as far south as Hazard Canyon, California (Goddard et al. 2023).

Here we more fully explore the diversity among *Hermissenda* species, which was first glimpsed by Lindsay et al. (2016) and Merlo et al. (2018) from mtDNA sequences. We sampled genome‐wide genetic variation from reduced‐representation genomes and used isolation‐with‐migration (IM) models to estimate the demographic history of divergences among these three species. We validated our demographic estimates with individual‐based evolutionary simulations using SLiM (Haller and Messer 2023). We explored possible reproductive isolation between the sympatric congeners *H. crassicornis* and *H. opalescens* through observations of mating behavior in laboratory experiments. Overall, we found large demographic differences and old divergence times (among all species) and little to no evidence of mating (between the sympatric species). The differences we document have interesting and general implications for understanding variation in the rate of divergence among different phenotypic traits, as well as specific implications for a possible role of species‐level differences in neurobiological or behavioral characteristics between *H. crassicornis* and *H. opalescens*.

## 2 Methods

### 2.1 Demographic Tests

Individuals of *Hermissenda crassicornis* and *H. opalescens* for ddRADSeq analysis were collected at shoreline locations in the northeastern Pacific (NEP) Ocean from southern British Columbia, Canada, down the coast to Baja California, Mexico in 2016 through 2018 (see Methods S1). Additional *H. emurai* sequences were obtained from individuals collected in the northwestern Pacific (NWP) Ocean during 2014 and at an unknown date in two locations in Japan and one in Russia. All individuals were identified to species by experts (ALKEP, AV) using morphological traits including the color and morphology of cerata. Processing was carried out from tissue samples from 46 nudibranchs at the Genomics Core Laboratory (Texas A&M University, Corpus Christi; see Methods S1). DNA was extracted from nudibranch tissue samples using the Omega BioTek EXNA DNA Isolation kit. The ddRAD library was constructed using a modified protocol from Peterson et al. (2012) and the dDocent pipeline (Puritz et al. 2014) was used for de novo assembly of the data. Each contig from that assembly was treated as a nonrecombining locus. Following sequence filtering to remove low confidence reads, sequencing artifacts, and parlogous loci, the rad_haplotyper application was used to infer the phases of single nucleotide polymorphisms (SNPs) in each locus and to generate haplotypes for each individual at every locus (Willis et al. 2017). Following assembly and filtering steps, a total of 2354 loci were obtained to be used in further demographic analyses.

Additional DNA samples of *H. crassicornis* and *H. opalescens* were also collected for mitochondrial DNA analysis (see Methods S1). These individuals were collected from several localities across the species' ranges. DNA was extracted using Chelex, DNEasy Blood and Tissue kits (Qiagen), and EZNA Mollusk kits (Omega Bio‐tak). Tissue samples were taken, and DNA extracted by the manufacturers' instructions. The resulting extraction supernatants were used for polymerase chain reaction (PCR) amplification confirmed using 1% agarose gel electrophoresis. We used the universal primers for the mitochondrial gene cytochrome *c* oxidase subunit I (*COI*) and additional primers designed for *Hermissenda*. PCR products were purified using the Thermo Scientific GeneJet purification kit (Waltham, MA) and primers diluted for sequencing. The resulting products, diluted primers, and purified samples were sent to Source Bioscience for Sanger sequencing (Santa Fe Springs, CA, USA).

The resulting *COI* sequences were combined with previously published sequences and the ddRADSeq loci to be used in demographic analyses. We used 65 *COI* sequences obtained from the National Center for Biotechnology Information (NCBI 1988) and the Barcode of Life Data System (accessed 14 March 2023) and 21 new *COI* sequences. The sequences were aligned (MUSCLE; Edgar 2004) in AliView (v. 1.26, Larsson 2014) and trimmed. Following filtering, a total of 73 sequences were used in subsequent analyses (see Table S1).

**FIGURE 1:**
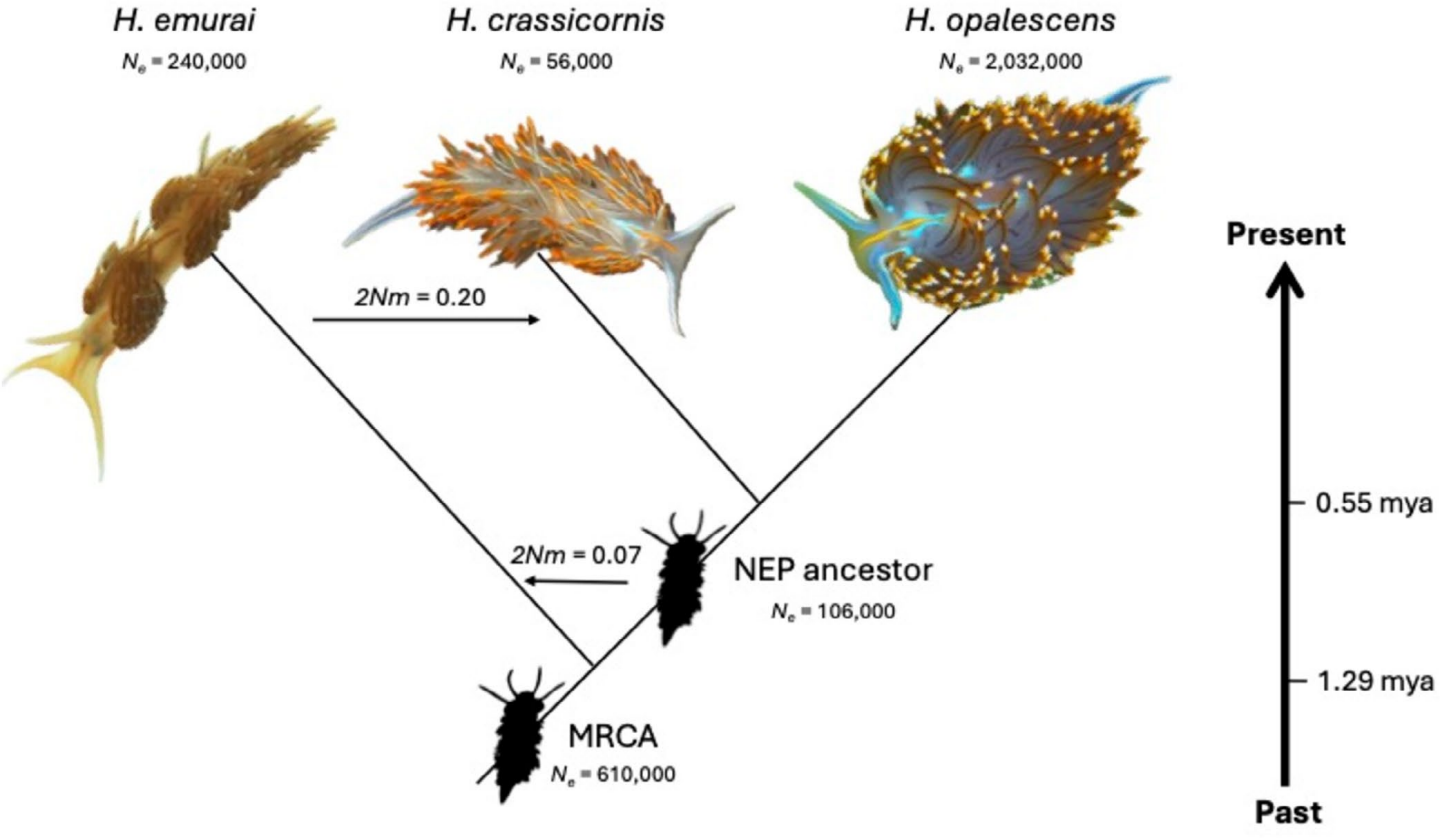
Summary of most likely phylogenetic tree topology and demographic parameter results of *Hermissenda* species, estimated from 2354 empirical loci and the IMa3 framework. Only the two highest 2*Nm* values are shown. 2*Nm* = population migration rate; MRCA, most recent common ancestor; *N*_e_, effective population sizes; NEP, northeastern Pacific.

We estimated demographic parameters for the three species using an isolation‐with‐migration (IM) Bayesian coalescent framework as implemented in IMa3 (Hey et al. 2018) running in parallel on the clusters operated by the Digital Research Alliance of Canada (alliancecan.ca; see Methods S1). Parameter values were estimated from models fit to the 2354 ddRADSeq loci plus the single mitochondrial *COI* locus using the Hasegawa‐Kishino‐Yano (HKY) mutation model (Hasegawa et al. 1985). We used a mutation rate calibration of 2.033 × 10^−5^ per gene per year for *COI* based on previous estimations of the lineage mutation rates for *Tridacna crocea* (Crandall et al. 2012). The three recognized *Hermissenda* species ( *H. crassicornis* , *H. emurai*, and *H. opalescens* ) and two ancestral groups (most recent common ancestor (MRCA) and NEP ancestor) were treated as populations in the analysis (Figure 1).

Prior to the estimation of demographic parameters, we first determined the most likely tree topology for the populations and used this for the remaining analyses (see Figure 1 for this topology). Appropriate prior distributions for demographic parameters in the model were determined using hyperprior distributions in IMa3 (see Supporting Information for an example of these priorfiles). We then modified these hyperpriors as necessary to estimate the 15 demographic parameters: two divergence times, five effective population sizes, and eight unidirectional gene flow rates among extant and ancestral populations. Due to computational intensity, we analyzed the loci in 12 batches, each with around 200 ddRADSeq loci and the *COI* locus (11 datasets with 100 ddRADSeq loci and one with 154 ddRADSeq loci; see in Supporting Information).

To validate the findings of the empirical data, we used the individual‐based evolutionary simulation framework SLiM (v. 4.01; Haller and Messer 2023) to simulate the same population history (with divergence times, population sizes, gene flow rates as estimated by IMa3 from the ddRADSeq data). We then sampled nucleotide sequences from these simulated populations and analyzed them in IMa3 (see Methods S1). Because these populations were large and simulating them was computationally intensive, we scaled the effective population sizes, divergence times, and mutation and recombination rates in our simulated model (as per Haller and Messer 2024) by 10‐fold. To estimate demographic parameters from simulated datasets, IMa3 was implemented on the high‐performance cluster (alliancecan.ca) as in the analysis of the ddRADSeq data. Please see the supplemental materials for the associated input file and simulation code.

### 2.2 Behavioral Tests

Because we found no evidence of gene flow between the two sympatric *Hermissenda* species, we examined courtship and mating interactions between and within those species. The experiment was conducted in summer 2017 at the Bamfield Marine Sciences Centre (BMSC), where both species had been abundant in nearby habitats in Barkley Sound and Clayoquot in summer 2016 (Merlo et al. 2018). Individuals of *Hermissenda opalescens* were scarce in Barkley Sound in 2017, and thus only *H. crassicornis* individuals (*n* = 56, approximate size range 1.5–4 cm) were collected locally, while *H. opalescens* individuals (*n* = 45, approximate size range 1.5–4 cm) were instead collected by MAC Bio‐Marine in Monterey Bay, CA, and shipped to BMSC. All animals were maintained in seawater at 12°C and 33 ppt with a natural photoperiod (approximately 16:8, light:dark), and fed every second day with fresh mussel carcasses (*Mytilus edulis*). *Hermissenda opalescens* were kept in self‐contained aquaria quarantined from the rest of the BMSC seawater system, but in otherwise similar conditions. All animal use protocols complied with the Canadian Council for Animal Care guidelines.

A series of experimental trials paired approximately size‐matched homospecific or heterospecific animals to test for differences in their behavioral interactions. Trials were conducted in mesh‐sided tubs (length × width × depth = 13 × 13 × 5 cm) in sea tables (large shallow fiberglass aquaria), with a water depth of 4 cm. The constant supply of flow‐through seawater in the sea table and mesh sides of the tubs ensured temperature, oxygenation, and other conditions throughout the trial were similar to the regular holding conditions. In each trial, two individuals were placed in randomly assigned opposite corners of a tub. Trials lasted 4 h, and were videoed at 2 frames per second by GoPro Hero 4 cameras fixed above the sea table (six tubs per sea table were simultaneously in view in each of 2 cameras, and thus 12 trials could be completed simultaneously). All tubs were washed between trials and tubs were positioned haphazardly within 2 rows of 3 tubs under each camera. A total of 96 trials were conducted during daylight hours on 5 different days, of which 92 provided usable data, divided among three treatments: *H. crassicornis* pairs (*n* = 28), *H. opalescens* pairs (*n* = 26), and heterospecific pairs (*n* = 38). As a consequence of the initial sample sizes and some mortalities during the experiment, we were not able to use each animal in only one trial. However, all pairs (the unit of replication) were unique, and individuals were used in either one (*n* = 54), two (*n* = 11), or, at most, three trials (*n* = 36). For those individuals used more than once, trials were separated by at least a day. All animals were euthanized following completion of the experiments.

Trial videos were reviewed in Fiji software (Schindelin et al. 2012) to quantify the frequency of three reproduction‐related behaviors. Rather than document the full complexity of all reproductive behaviors described in previous reports, we focused on three that were clearly observable in our videos (Table 1; Zack 1975; Longley and Longley 1982; Rutowski 1982): flagellation (usually occurs prior to copulation), copulation (recognized by ceratal movements), and aggression (an additional potential outcome observed for conspecific interactions in *H. crassicornis* ). Durations of each occurrence of each behavior were used to count the number of trials with at least one occurrence (total behavior duration for the trial > 0), and for each behavior, those trial counts were compared between pair types with chi‐squared tests. For a more detailed comparison, durations were converted to proportions of the total trial duration for each behavior, and each analyzed separately using an aligned rank transform nonparametric single factor model (v. 4.2.2; R Core Team 2023, ARTool package; Elkin et al. 2021; Wobbrock et al. 2011) comparing the effect of the three pair types (two homospecific, one heterospecific). Although some individuals were used more than once, we chose not to include random effects for individuals in our models based on the following rationale. First, pairs were the unit of replication in the study, and all pairs were unique. Second, each pair included two individuals, which would require two separate random effects for the 1st and 2nd member of each pair, resulting in duplicated random effects for some individuals (e.g., if pairs tested included individuals 1 and 2, 2 and 3, and 3 and 4, it is impossible to design a model with a single random effect for each individual since both individuals 2 and 3 would be included in the random effect terms for both the 1st and 2nd members of each pair). Nonetheless, mixed effect models (which are possible with the ARTool package) with two random effects for the 1st and 2nd members of each pair were attempted, but produced errors in the model fitting because they had too many parameters to estimate given our total replication. Given that the individuals were included in a trial at most three times out of 96 trials, the risk of bias in our results arising from this level of pseudoreplication is low, and in the absence of a workable model with random effects, the pragmatic approach is to not include them in the analysis (Colegrave and Ruxton 2018).

**TABLE 1:** Reproduction‐related behaviors quantified between homospecific and heterospecific pairs. Behaviors in italics are described in Zack (1975), with further details regarding recognition of copulation from Longley and Longley (1982) and Rutowski (1982).

Behavior	Description
Flagellation	Pair positioned anterior‐to‐anterior, with repeated mutual touching of oral tentacles; typically occurs prior to *sidling* (forward movement along the right side of the other slug) and then copulation.
Copulation	With heads overlapping right‐side‐to right‐side (this aligns the gonopores), both animals raise cerata (“*cerata movement*”), which acts a distinctive sign that copulation is likely occurring. Typically follows *sidling*.
Aggression	Aggressive behavior between slug pairs that lasts for longer than 4 s, including *biting* and *lunging*. These behaviors are alternate potential outcomes of conspecific encounters rather than copulation.

## 3 Results

### 3.1 Demographic Tests

A total of 11 *H. crassicornis* , 27 *H. opalescens*, and 8 *H. emurai* individuals were used in ddRADSeq library preparation. The raw data across species contained 99,362 contigs with a mean length of 296 bps. These were filtered to 96,458 contigs that were positively identified to the species level. After further filtering for quality control, the assembly included 2354 contigs (mean length of 256 base pairs) from 33 individuals (9 from *H. crassicornis*, 7 from *H. emurai*, and 17 from *H. opalescens* ) with a mean of 10.8 SNPs per contig and 25,350 SNPs total.

A total of 73 *COI* sequences were retained (out of 90 available sequences) following quality control (based on sequence length, certainty of identification) to be used in demographic analyses: 39 *H. crassicornis* , 7 *H. emurai*, and 27 *H. opalescens* sequences (Table S1). An IM model was fit to the 2354 ddRADSeq loci and the single *COI* locus to estimate the phylogenetic tree topology. The most likely tree topology (posterior probability = 0.7452) placed *H. crassicornis* and *H. opalescens* as the sister species. The next most likely species tree had *H. emurai* and *H. opalescens* as the sister species (posterior probability = 0.2548).

**FIGURE 2:**
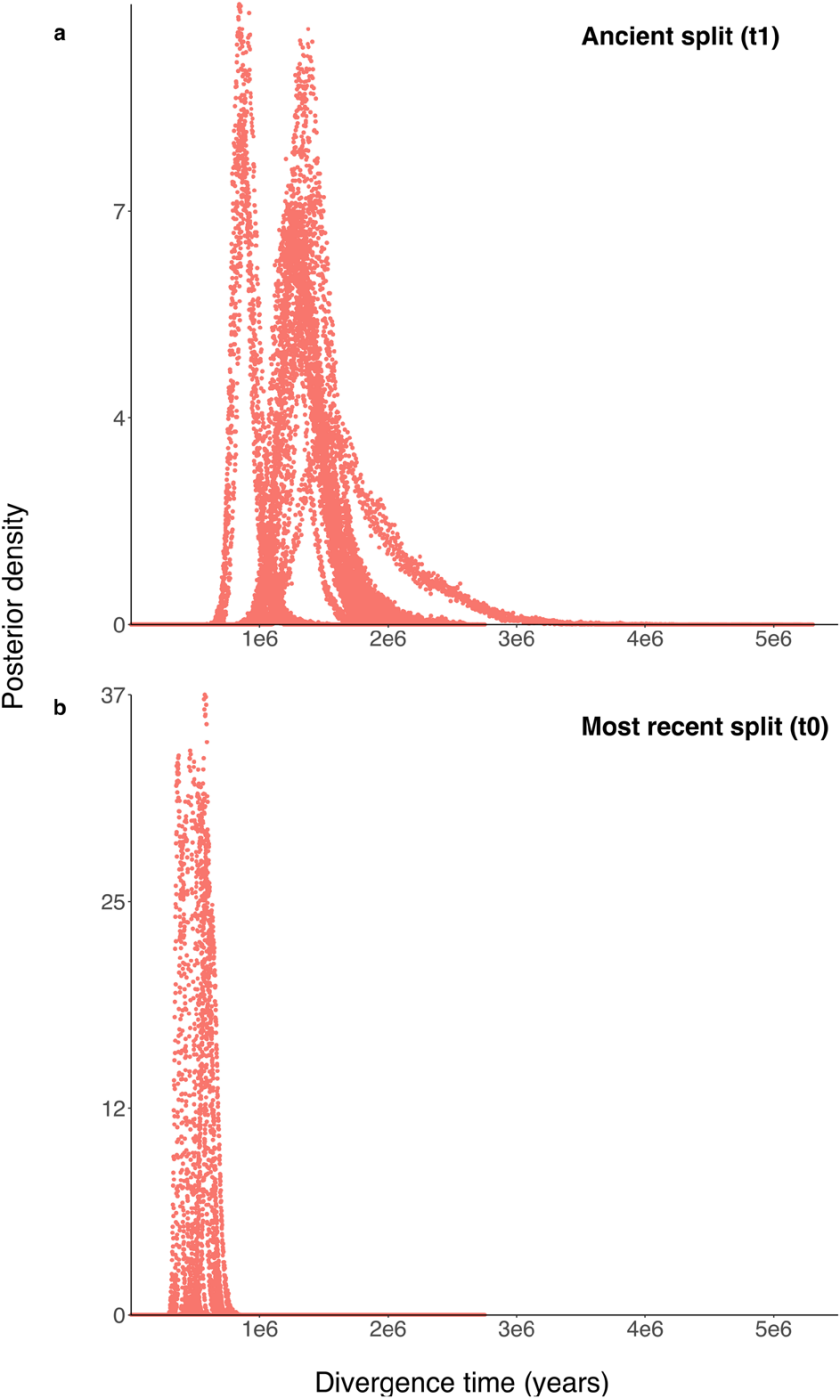
The posterior probability distributions for divergence times (in years) within the genus *Hermissenda* estimated using IMa3. The top panel (a) represents the basal split to form an ancestral northeastern population and northwestern *H. emurai*; the bottom panel (b) shows the more recent split creating the northeastern populations *H. crassicornis* and *H. opalescens* . Each colored distribution represents one of the 24 model runs (12 sets of 200 loci run in duplicate).

**FIGURE 3:**
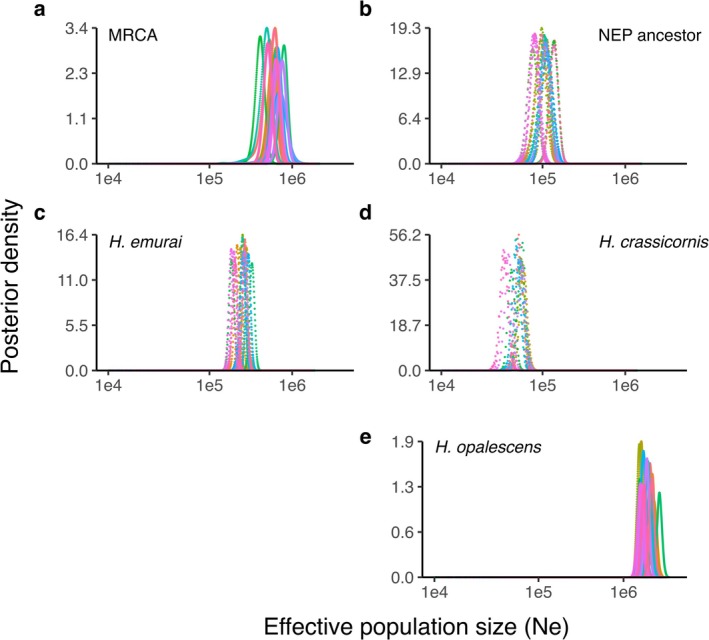
The posterior probability distributions of effective population sizes of ancestral and extant populations in the genus *Hermissenda* estimated using IMa3. Colors represent 24 independent model runs. (NEP = northeastern Pacific ancestral population, MRCA = most recent common ancestor for all *Hermissenda* species).

**FIGURE 4:**
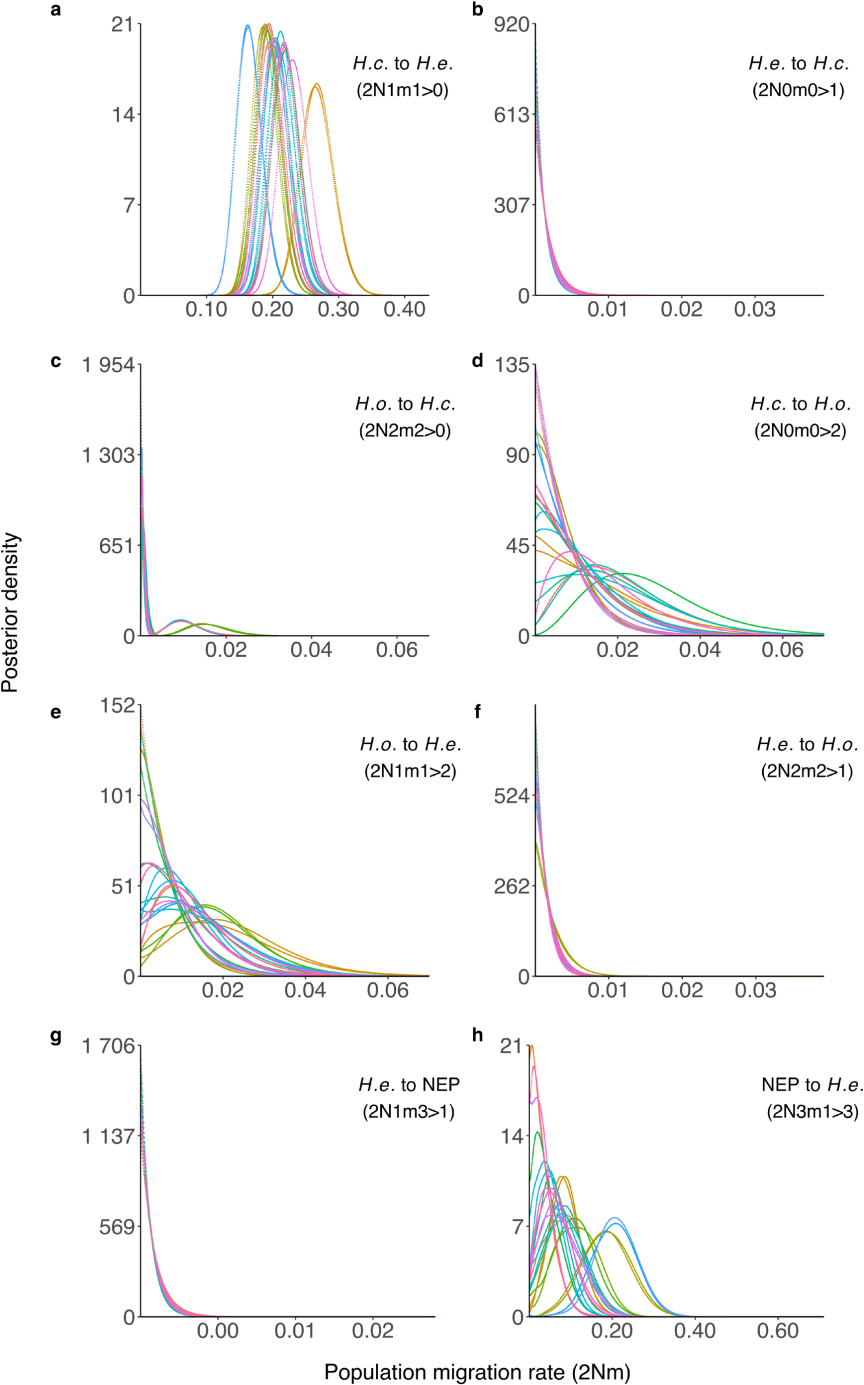
The posterior probability distributions for population migration rates (2*Nm*) between populations in the *Hermissenda* genus estimated using IMa3, labeled using IMa3 notation. Migration direction in coalescent time is indicated in each panel. Colors represent independent model runs. *H.c*., *H. crassicornis* ; *H.e*., *H. emurai*; *H.o*., *H. opalescens* ; NEP, northeastern Pacific ancestral population.

In all other analyses, we used that most‐likely species tree as the input tree and used IMa3 to estimate the 15 demographic model parameters for the three *Hermissenda* species: two divergence times (*t*), five effective population sizes (*N*_e_), and eight population migration rates (2*Nm*; Figure 1; Table 2). The median values of the most‐likely parameter estimates and the lower and upper bounds of the 95% highest posterior densities across all 24 duplicate analyses for 12 datasets are shown in Tables S2 and S3. The first split between the eastern and western lineages was estimated at 1.29 mya (Figure 2a), in the early Pleistocene. The more recent split, between *H. crassicornis* and *H. opalescens* , is estimated to have taken place 0.55 mya (Figure 2b) in the mid‐Pleistocene. Effective population sizes decreased after the first speciation event from *N*_e_ > 600,000 in the most recent common ancestor (Figure 3a) to approximately 100,000–250,000 in the eastern and western Pacific lineages (Figure 3b,c). After the second speciation event, *N*_*e*_ decreased in northern *H. crassicornis* (*N*_e_ < 60,000; Figure 3d) but greatly increased in southern *H. opalescens* (*N*_e_ > 2,000,000; Figure 3e), which consistently was estimated to be larger than all other historical or ancestral lineages in the model. We found one relatively high population migration (or hybridization) rate: west‐to‐east gene flow from *H. emurai* into *H. crassicornis* (2*Nm* ~ 0.2, or approximately one immigrant gene copy every fifth generation; Figure 4c). Most other population migration rates were very low (2*Nm* < 5 × 10^−4^; Figure 4). The rate of ancient east–west migration from the Northeastern Pacific lineage into *H. emurai* (2*Nm* ~ 0.07; Figure 4a) was also estimated to be slightly higher but with posterior distributions that varied widely among datasets and included nonzero probabilities for 2*Nm* = 0. These output files are available in the Supporting Information.

**TABLE 2:** The most likely demographic parameter estimates from empirical and simulated datasets for divergence times (*t*), effective population sizes (*N*_e_), and population migration rates (2*Nm*).

Parameter	Empirical	Simulated
*t*_0_	1,290,000	1,965,223
*t*_1_	550,000	582,627
*q*_0_	56,000	64,055
*q*_1_	240,000	312,125
*q*_2_	2,032,000	2,636,596
*q*_3_	106,000	157,483
*q*_4_	610,000	398,880
*2Nm*_1>0_	0.20	0.0007
*2Nm*_1>3_	0.07	0.00003

*Note:* Empirical data come from 24 IMa3 output files (12 datasets of ~200 loci each duplicated) and simulated from two output files (1 dataset of 200 loci duplicated). The estimate *t*_0_ represents the most recent split in the northeastern Pacific to create *H. crassicornis* and *H. opalescens* ; *t*_1_ represents the most ancestral split to create the northeastern Pacific ancestor and *H. emurai*. The *N*_e_ estimates numbered 0–4 represent *Hermissenda crassicornis* , *H. emurai*, *H. opalescens* , the northeastern Pacific ancestor, and the most recent common ancestor for all populations, respectively. 2*Nm* rates are understood as the rate by which genes of a population are supplanted by genes from another. Only the two highest (of 8) migration rates from the empirical datasets are shown, all others can be found in the Supporting Information. These numbers are rounded for clarity.

We then simulated a dataset using these estimated parameters (Table 2; Tables S4 and S5) and asked if IMa3 could re‐estimate parameter values from the simulated data that were similar to parameter values from the empirical data. Under the 10‐fold scaled model, in which all input parameters of the simulation were set to the scaled median values from our empirical data, IMa3 inferred a first population split approximately 1.97 mya and a second split to create populations representing *H. opalescens* and *H. crassicornis* approximately 0.58 mya (Figure S1). The most ancestral population was inferred to be > 400,000 individuals (Figure S2a) followed by decreases in *N*_*e*_ for both the eastern ancestor at approximately 157,000 individuals (Figure S2b) and *H. emurai* with approximately 310,000 individuals (Figure S2c) after the first speciation event. Following the second speciation event in the east, the northern *H. crassicornis N*_*e*_ decreased further (64,000 individuals; Figure S2d) whereas the southern *H. opalescens* increased substantially (> 2,600,000 individuals; Figure S2e). Those results were broadly similar to the empirical values used in the simulation. All migration rates were estimated to be low in this simulation, with the largest rate being east‐to‐west gene flow from *H. crassicornis* into *H. emurai* (2*Nm* = 0.007, Figure S3d). This is a similar finding to the empirical results, but the highest gene flow rate in the empirical data was found in the other direction (west‐to‐east gene flow from *H. emurai* into *H. crassicornis* ; Figure 1). These output files are available in the Supporting Information.

### 3.2 Behavioral Tests

**FIGURE 5:**
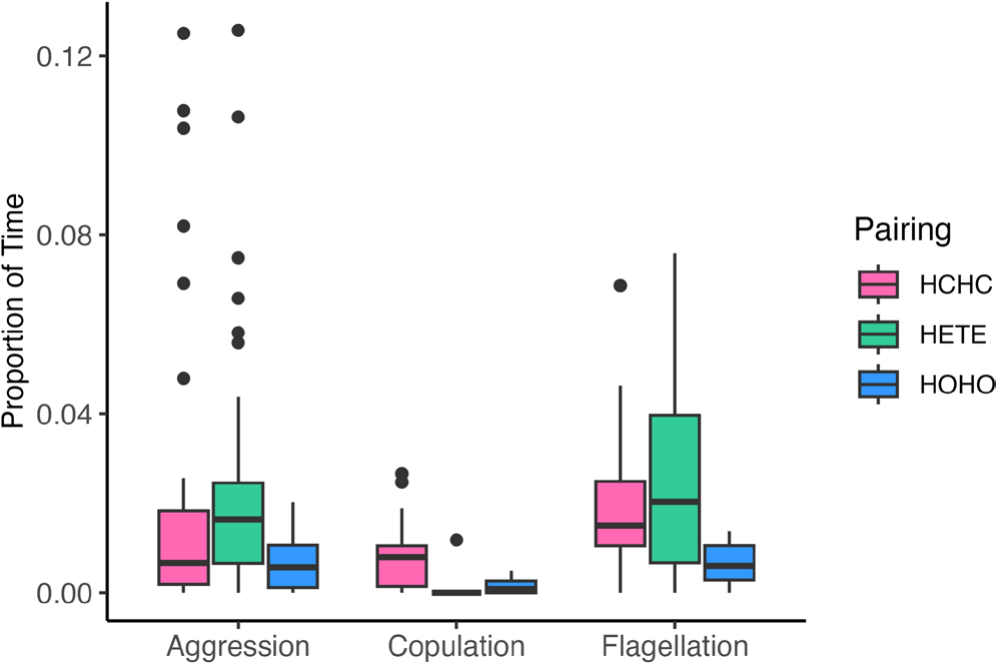
Proportion of time during mating trials spent on three different reproductive behaviors for homospecific ( *Hermissenda crassicornis* = HCHC or *Hermissenda opalescens* = HOHO) and heterospecific (HETE) *Hermissenda* species pairs. Points represent outliers and whiskers indicate the highest and lowest values within 1.5× the interquartile range.

Reproductive behavior clearly differed among homospecific and heterospecific pairs. Most importantly, 37 of 38 heterospecific pairs never performed copulation (Table 3). Heterospecific pairs would begin the mating sequence, starting with flagellation and sidling, but interactions typically ended in aggression without copulation. In a single outlier trial, three behaviors were observed that met our criteria for copulation, but we are not certain the animals actually copulated in this case. In comparison, both types of homospecific pairs were frequently observed apparently copulating (Table 3). By contrast, both flagellation and aggression behaviors were frequently observed in both homospecific and heterospecific pairs (Table 3; see also Supporting Information (“behavioral” folder) for an example video of these behaviors). Proportions of each trial spent on the three behaviors were relatively low, rarely exceeding 10% of the time. Unsurprisingly, the proportion of time spent copulating was significantly higher for both homospecific pairs than for heterospecific pairs (Figure 5; Table 4). Homospecific *H. opalescens* pairs spent significantly less time performing flagellation than *H. crassicornis* pairs and heterospecific pairs (Figure 5; Table 4). Homospecific *H. opalescens* pairs also exhibited significantly less aggression than heterospecific pairs, but not homospecific *H. crassicornis* pairs (Figure 5; Table 4).

**TABLE 3:** Proportions of trials with at least one occurrence of reproductive behaviors for three pair types (two homospecific = HCHC ( *Hermissenda crassicornis* ) and HOHO (*Hermissenda opalescens*), one heterospecific = HETE).

Behavior	HCHC (*n* = 28)	HETE (*n* = 38)	HOHO (*n* = 26)	*p*
Copulation	75%^a^	3%^b^	50%^a^	< 0.0001
Flagellation	93%	79%	96%	0.073
Aggression	82%	97%	85%	0.099

*Note:* For each behavior, *p‐*values are provided for a chi‐square test comparing the pair types; significantly different pair types from post hoc pairwise chi‐square tests are indicated by different superscript letters.

**TABLE 4:** Aligned rank transform nonparametric single factor models testing the effect of the three pair types (two homospecific = HCHC ( *Hermissenda crassicornis* ) and HOHO (*Hermissenda opalescens*), one heterospecific = HETE) on three reproductive behaviors.

Behavior	df	SS	*F*	Pr > *F*	HCHC	HETE	HOHO
Copulation	2	22111.63	35.99	3.53E^−12^	a	b	c
Flagellation	2	10664.60	8.77	3.34E^−4^	a	a	b
Aggression	2	7510.63	5.83	4.16E^−3^	ab	a	b

*Note:* Letters in the final three columns indicate homogeneous subsets of treatments following post hoc comparisons.

## 4 Discussion

Demographic analyses can provide crucial information linking ecological and evolutionary processes across spatial and temporal scales (see Griffith et al. 2016 for a discussion). In this study, we first conducted demographic analyses to infer the evolutionary history of the nudibranch genus *Hermissenda*. We found support for a new branching pattern (see Lindsay and Valdés (2016) for previous topology) for the *Hermissenda* genus, in which the sympatric species *H. crassicornis* and *H. opalescens* are the sister species. We further estimated (1) divergence times in the mid‐ to late Pleistocene (1.29–0.55 mya), (2) a much larger effective population size of *H. opalescens* than of other species, and (3) limited gene flow among all species in the genus. Our behavioral data showing no mating between sister species are consistent with this latter conclusion.

The *Hermissenda* genus phylogeny had previously been inferred from two mitochondrial (*COI* and *16S*) and two nuclear (*H3* and *18S*) gene fragments (Lindsay and Valdés 2016), and suggested that the two northern clades are sister groups. However, most loci in the ddRADSeq data supported the topology shown in Figure 1. This type of discordance between gene trees is unsurprising: gene trees sampled from closely related species or populations are expected to vary among loci (Richards et al. 2018).

We estimated that the first divergence event in the *Hermissenda* genus to have occurred approximately 1.29 mya, in the late Pleistocene, giving rise to an eastern and a western lineage. The more recent divergence event, creating *H. opalescens* and *H. crassicornis* in the NEP, took place 0.55 mya, in the mid‐Pleistocene. These times seem old relative to the modest morphological differences between the congeners and the readily observable morphological differences between congeneric species that are estimated to have diverged on shorter time scales (Singhal et al. 2018). For example, the morphologically distinct congeners *Nucella ostrina* and *N. emarginata* diverged as recently as 1 mya (Marko 1998; Marko et al. 2014). However, sister taxa can also be very similar (at the limit, cryptic species pairs) because of morphological stasis brought about by low standing genetic variation, developmental constraints, or environmental drivers that cause stabilizing selection (Struck et al. 2018).

In our analyses, we found that the two eastern sympatric species had vastly different inferred *N*_e_ values, with the southern *H. opalescens* population having an approximately 30× larger *N*_e_ than its northern congener *H. crassicornis* (> 2,000,000 and < 60,000 individuals, respectively). All other estimates were much smaller (with a most recent common ancestral population ~600,000 individuals). This difference implies a large population expansion in the southern portion of the NEP following the divergence event 0.55 mya in this region. It is possible that during intense glaciation in the Pleistocene, *H. opalescens* individuals were able to survive at large population sizes in the more southern part of the *Hermissenda* range. In comparison, *H. crassicornis* individuals in the north may have had to retreat to glacial refugia during glacial periods, or may be descended from the leading edge of the northern distribution of *H. opalescens* . While not a direct measure of census size, low *N*_e_ may indicate smaller overall population sizes (see Nadachowska‐Brzyska et al. 2022 for review). Importantly, all *N*_e_ estimates were relatively large (thousands of individuals). Although the number of individuals for most nudibranch species is unknown, these estimates seem to indicate healthy population sizes. Because the IMa3 model assumes a constant population size before and after divergence events (Hey 2005), population trends after the split between *H. crassicornis* and *H. opalescens* are unknown.

Most gene flow rates between *Hermissenda* species were low (up to 2*Nm* ~ 0.2). For context, a population migration rate of 10 migrants per generation is often considered to be enough to prevent divergence between demes, and a rate of 1 migrant per generation can limit the loss of genetic diversity within demes (Lowe and Allendorf 2010). This modest estimated migration rate across the North Pacific may be the result of small amounts of contemporary stepping‐stone gene flow between *H. emurai* and *H. crassicornis* populations in the Aleutian Islands where these species could be sympatric, though their northern ranges are currently undocumented.

In our simulated dataset, the inferred demographic parameter values of the simulated model were largely consistent with our empirical data (Table 2). However, we found some modest differences including a first divergence time in the simulated data that was inferred to be earlier (1.97 mya) than the empirical estimate (1.29 mya) used in the simulation. Similarly, we found some gene flow from *H. crassicornis* into *H. emurai* in the empirical data (2*Nm* ~ 0.2), but this gene flow was not detected in IMa3 analyses of the simulated data, where all rates of gene flow were low (2*Nm* < 0.007). These differences suggest that IMa3 may be slightly overestimating the real divergence times or underestimating the low rates of gene flow between *Hermissenda* species in the empirical analyses of the ddRADSeq data. However, a second possible cause of the discrepancy between empirical and simulated results may come from not IMa3, but from SLiM, specifically due to the scaling used in the simulated model. Although scaling is recommended by the creators of the SLiM software (Haller and Messer 2024), caution is needed. In models scaled down to very small size, the stochastic effects of a few mutations can be disproportionately large due to the decreased number of total mutations (Haller and Messer 2024). Rescaling by a factor of 10 may seem large but scaling Wright‐Fisher simulations in SLiM in this range has been shown to have little to no effect on the normalized site frequency spectrum (Cury et al. 2022). By contrast, some studies have shown that rescaling by a factor of 50 influences patterns of sequence diversity and linkage disequilibrium that might have a detectable effect on population genetic variation sampled from the simulated population (Adrion et al. 2020). It seems likely that our model scaling accounts for these small discrepancies between the simulation results and the empirical parameter values we used in the simulations. We are confident that the empirical results from analysis of the ddRADSeq data reflect real old divergences among species and reproductive isolation between the two sympatric species.

Our findings suggest complete reproductive isolation between the sister species *H. crassicornis* and *H. opalescens* . Although the species are sympatric from southern British Columbia, Canada to central California, USA (Lindsay and Valdés 2016; Goddard et al. 2023), gene flow rates near zero indicate one or more strong isolating mechanisms. The experimental results in which heterospecifics were not observed to copulate in the lab suggest a prezygotic barrier from assortative mating by species. Given the consistency of findings across all other trials, we are inclined to consider the single case of heterospecific copulation observed as experimental error from using ceratal movements as indicative of mating rather than direct observation of copulation itself. We do note that *H. opalescens* were imported for the experiment, and thus either transportation stress or differing environmental or holding conditions prior to the experiment could have limited heterospecific copulation that might otherwise occur in natural habitats. Nonetheless, since approximately half of *H. opalescens* pairs exhibited apparent copulation with other *H. opalescens* and effectively none was observed with *H. crassicornis*, we conclude for now that the two species do not (or are unlikely to) mate with each other, contributing to the species separation (Coyne and Orr 2004). The exact barrier may be chemical or physiological, preventing heterospecifics from being recognized as potential mates (Zack 1975; Paterson 1980; Palumbi 1994). Behavioral or sensory differences that impede compatible reproductive movements between species could also contribute to reproductive isolation (Palumbi 1994; Coyne and Orr 2004), as well as a mechanical barrier, such as differences in reproductive anatomy like those seen in *Glaucus atlanticus* and *G. marginatus* (Coyne and Orr 2004; Churchill et al. 2013). A more detailed analysis of the various behaviors involved: flagellation, lunging, biting, sidling, copulation itself, ceratal movement, etc. (Zack 1975; Longley and Longley 1982; Rutowski 1982) for both homospecific and heterospecific pairs collected from other sympatric locations could further verify the findings here and add insight into the nature of the prezygotic barrier between these two species.

Beyond the morphological and genetic differences identified to date, other differences between the two species may exist. Reproductively isolated species that use similar resources (Mayr 1947; Howard and Berlocher 1998) may compete if their niches overlap (Wissinger 1992), or competition could be reduced through the evolution of resource partitioning (Pianka 1974; Sinopoli et al. 2017). Unfortunately, little is known about any ecological differences across the ranges of either *H. crassicornis* and *H. opalescens*. Our observations of short duration copulation (when it occurred) and both flagellation and aggression between homospecific pairs are all consistent with past reports (Zack 1975; Longley and Longley 1982; Rutowski 1982). In our experiment, *H. crassicornis* was more aggressive than its congener, potentially giving it a competitive advantage. However, there was also some evidence of overall lower levels of motivation to reproduce by *H. opalescens* (less copulation and flagellation). This difference could be innate to the two species, a consequence of age or other characteristic of the two specific populations sampled or linked to the differing holding conditions and stress arising from *H. opalescens* being collected and shipped from California to British Columbia. Further experiments are now needed to test for other niche differences between species, particularly diet preferences, which preliminary observations suggested may differ (Estores‐Pacheco 2020).

Determining the mechanism of speciation is probably not possible over a time scale of 10^5^–10^6^ years for *Hermissenda* nudibranchs with large effective population sizes that seem to be completely reproductively isolated. In the Pacific Ocean, several examples of cryptic speciation have been associated with the last glacial maximum (Mix et al. 2001; Clark et al. 2009; Ekimova et al. 2023). More generally, glacial cycles throughout the Pliocene and Pleistocene are thought to have facilitated the speciation of many marine species (Hewitt 2000), including some nudibranchs in the northeastern Pacific (Lindsay et al. 2016). It is possible that the *Hermissenda* species complex diverged in a similar way, associated with cyclical bouts of vicariance followed by dispersal during glacial–interglacial cycles (Lindsay et al. 2016). An alternative hypothesis is sympatric speciation between *Hermissenda crassicornis* and *H. opalescens* . Our finding of apparently strong prezygotic isolation between those species is consistent with widespread observations of stronger prezygotic isolation between sympatric than between allopatric species pairs (Coyne and Orr 1989) due to the effects of reinforcement, and may indicate sympatric speciation between them, rather than allopatric speciation followed by secondary contact during the current interglacial period.

The discovery of previously uncharacterized differences among the species may impact the interpretation of previous neurobiological findings from *Hermissenda* species. *Hermissenda crassicornis* (sensu lato) has been the subject of a variety of neurobiological and behavioral studies (e.g., Alkon 1973; Crow and Alkon 1980; Blackwell 2006; Gunaratne and Katz 2016; Webber et al. 2017). However, we must now consider the possibility that differences in neuroanatomy, neurophysiology, or behavior may exist among the species, especially in light of the significant differences among species in levels of aggression. In particular, we suggest three further important steps. Clearly, future work needs to identify which species is being studied, along with collection location, and ideally, mitochondrial haplotyping. In addition, a retrospective review is needed to categorize findings by the (likely) species studied and generate hypotheses about species commonalities and differences. Finally, specific comparisons of particular neurobiological or other biological findings across *Hermissenda* species would help to assess the extent of the potential problem with past findings and may yield insights into the possible factors driving speciation in marine systems.

## Author Contributions

**Miranda T. Dennis:** conceptualization (equal), data curation (lead), formal analysis (lead), investigation (equal), methodology (equal), project administration (lead), resources (equal), software (lead), supervision (equal), validation (lead), visualization (lead), writing – original draft (lead), writing – review and editing (equal). **Austin L. Ka’ala Estores‐Pacheco:** conceptualization (equal), data curation (supporting), investigation (equal), methodology (equal), resources (equal), validation (supporting), writing – original draft (supporting). **Keilan Williams:** conceptualization (supporting), methodology (supporting). **Russell C. Wyeth:** conceptualization (supporting), formal analysis (equal), methodology (equal), software (equal), supervision (supporting), validation (supporting), visualization (supporting), writing – original draft (supporting), writing – review and editing (supporting). **Ángel Valdés:** conceptualization (equal), data curation (supporting), formal analysis (supporting), investigation (supporting), methodology (equal), project administration (equal), resources (equal), software (equal), supervision (equal), validation (equal), writing – review and editing (supporting). **Arne Ø. Mooers:** conceptualization (equal), formal analysis (supporting), funding acquisition (equal), investigation (equal), methodology (equal), project administration (equal), supervision (equal), validation (supporting), visualization (supporting), writing – original draft (supporting), writing – review and editing (supporting). **Michael W. Hart:** conceptualization (equal), data curation (supporting), formal analysis (supporting), funding acquisition (equal), investigation (supporting), methodology (equal), project administration (equal), resources (equal), software (supporting), supervision (equal), validation (supporting), visualization (supporting), writing – original draft (supporting), writing – review and editing (supporting).

## Funding

Funded by Natural Science and Engineering Research Council of Canada Discovery Grants to A.Ø.M., M.W.H., and R.C.W. The funders had no role in study design, data collection and analysis, decision to publish, or preparation of the manuscript.

## Ethics Statement

Animal collection and care for mating experiments was approved by the Bamfield Marine Sciences Centre (BMSC) animal care committee (animal use protocol RS‐17‐06).

## Conflicts of Interest

The authors declare no conflicts of interest.

## Supporting information


**Data S1:** ece373045‐sup‐0001‐Supinfo.docx.

## Data Availability

The data that support the findings of this study are openly available in Mendeley Data, V3, https://data.mendeley.com/datasets/4rzn3hkpcy/3 (https://doi.org/10.17632/4rzn3hkpcy.3).
