# Supplementary material for: Demographic Models and Behavioral Assessments Uncover Distinct Species Histories in the Pseudocryptic Nudibranch Genus Hermissenda
Source: Ecol Evol. 2026 Feb 3;16(2):e73045. doi: 10.1002/ece3.73045 (PMC12867953; doi:10.1002/ece3.73045)
Supplement: Supplementary file 1 — Data S1: ece373045‐sup‐0001‐Supinfo.docx. [file ECE3-16-e73045-s001.docx]

**Supplemental Tables and Figures for:**

**Demographic models and behavioural assessments uncover distinct species histories in the pseudocryptic nudibranch genus *Hermissenda***

Dennis, Miranda T., Estores-Pacheco, A. L. Ka’ala, Williams, Keilan, Wyeth, Russell C., Valdés, Ángel., Mooers, Arne Ø., and Hart. Michael W.

**Table of Contents:**

| **Table S1** | Page 2-4 |
| --- | --- |
| **Table S2** | Page 5-6 |
| **Table S3** | Page 7-9 |
| **Table S4** | Page 10 |
| **Table S5** | Page 11 |
| **Figure S1** | Page 12 |
| **Figure S2** | Page 13 |
| **Figure S3** | Page 14 |

**Supplementary Tables and Figures**

Table S1. Information for cytochrome c oxidase subunit I sequences of *Hermissenda* nudibranchs included in all input files for estimation of most likely tree topology and demographic parameters in IMa3.

| Species | Location | GenBank Accession Number |
| --- | --- | --- |
| *Hermissenda crassicornis* | No info | JQ699630 |
|  | No info | MH304626 |
|  | Cook Inlet, Alaska, USA | KF643647 |
|  | Sitka, Alaska, USA | KU950165 |
|  | Sitka, Alaska, USA | KU950166 |
|  | Northeast Pacific, British Columbia, Canada | MH235587 |
|  | Northeast Pacific, British Columbia, Canada | MH235593 |
|  | Northeast Pacific, British Columbia, Canada | MH235594 |
|  | Central Coast, British Columbia, Canada | MH235588 |
|  | Central Coast, British Columbia, Canada | MH235589 |
|  | Central Coast, British Columbia, Canada | MH235590 |
|  | Central Coast, British Columbia, Canada | MH235591 |
|  | Central Coast, British Columbia, Canada | MH235592 |
|  | Barkley Sound, British Columbia, Canada | MH235595 |
|  | Barkley Sound, British Columbia, Canada | MH235597 |
|  | Barkley Sound, British Columbia, Canada | MH235599 |
|  | Bamfield, British Columbia, Canada | MH235600 |
|  | Tsawwassen, British Columbia, Canada | MG421500 |
|  | Victoria, British Columbia, Canada | KU950167 |
|  | Victoria, British Columbia, Canada | KU950168 |
|  | Victoria, British Columbia, Canada | KU950169 |
|  | Victoria, British Columbia, Canada | KU950170 |
|  | Victoria, British Columbia, Canada | KU950171 |
|  | Victoria, British Columbia, Canada | KU950172 |
|  | Victoria, British Columbia, Canada | KU950173 |
|  | Victoria, British Columbia, Canada | KU950174 |
|  | Victoria, British Columbia, Canada | KU950175 |
|  | Victoria, British Columbia, Canada | KU950176 |
|  | Victoria, British Columbia, Canada | KU950177 |
|  | San Juan County, Washington, USA | MH242795 |
|  | Jefferson County, Washington, USA | BBPS820-19* |
|  | Gig Harbor, Washington, USA | KU950160 |
|  | Gig Harbor, Washington, USA | KU950161 |
|  | Lane County, Oregon, USA | KU950163 |
|  | Humboldt Bay, California, USA | KU950178 |
|  | Bodega Bay, California, USA | OQ427258 |
|  | Bodega Bay, California, USA | OQ427259 |
|  | Point Reyes, California, USA | KU950164 |
|  | Monterey Bay, California, USA | KU950179 |
| *Hermissenda opalescens* | No info | KX889736 |
|  | Barkley Sound, British Columbia, Canada | MH235596 |
|  | Whiskey Creek, Oregon, USA | OQ427269 |
|  | Bodega Bay, California, USA | KU950190 |
|  | Bodega Bay, California, USA | KU950191 |
|  | Bodega Bay, California, USA | OQ427271 |
|  | Monterey Bay, California, USA | KU950196 |
|  | Morro Bay, California, USA | OQ427265 |
|  | Morro Bay, California, USA | OQ427266 |
|  | Morro Bay, California, USA | OQ427274 |
|  | Avila Beach, California, USA | OQ427263 |
|  | Avila Beach, California, USA | OQ427272 |
|  | Santa Barbara, California, USA | OQ427268 |
|  | Santa Barbara, California, USA | OQ427270 |
|  | Santa Barbara, California, USA | OQ427275 |
|  | Santa Barbara, California, USA | OQ427277 |
|  | Ventura County, California, USA | OQ427267 |
|  | Malibu, California, USA | KU950195 |
|  | Malibu, California, USA | OQ427262 |
|  | Malibu, California, USA | OQ427264 |
|  | Malibu, California, USA | OQ427273 |
|  | Long Beach, California, USA | KU950192 |
|  | Long Beach, California, USA | KU950193 |
|  | Long Beach, California, USA | KU950194 |
|  | Orange County, California, USA | GASSC569-18* |
|  | Bahia de Los Angeles, Mexico | KU950187 |
|  | Bahia de Los Angeles, Mexico | KU950189 |
| *Hermissenda emurai* | Muroran, Hokkaido, Japan | KU950180 |
|  | Muroran, Hokkaido, Japan | KU950181 |
|  | Muroran, Hokkaido, Japan | KU950182 |
|  | Muroran, Hokkaido, Japan | KU950183 |
|  | Muroran, Hokkaido, Japan | KU950184 |
|  | Tateyama, Chiba, Japan | KU950185 |
|  | Tateyama, Chiba, Japan | KU950186 |

Table S2. The most likely demographic parameter value estimates (max) and the values of the limits on the associated 95% highest posterior density interval (lower, upper) for divergence times (t) and effective population sizes (N_e_) from 24 IMa3 output files (12 datasets of ~200 loci each duplicated). The estimate t_0_ represents the most recent split in the northeastern Pacific to create H. crassicornis and H. opalescens; t_1_ represents the most ancestral split to create the northeastern Pacific ancestor and H. emurai. The N_e_ estimates numbered 0-4 represent Hermissenda crassicornis, H. emurai, H. opalescens, the northeastern Pacific ancestor, and the most recent common ancestor for all populations, respectively.

| Dataset | Duplicate | Estimate | *t_0_* | *t_1_* | *q_0_* | *q_1_* | *q_2_* | *q_3_* | *q_4_* |
| --- | --- | --- | --- | --- | --- | --- | --- | --- | --- |
| 1 | 1 | Max  Lower  Upper | **566300**  496019 636581 | **1277216**  1047452 1709713 | **55575**  37285 73866 | **254520**  218867 296114 | **2237804**  1922316 2584223 | **110959**  80182 148215 | **510424**  344507 694775 |
|  | 2 | Max  Lower  Upper | **523065**  466794 599799 | **1205992**  980907 1656160 | **52587**  35280 69894 | **238962**  205226 278320 | **2099933**  1816040 2439434 | **104993**  74338 138714 | **472079**  319444 637798 |
| 2 | 1 | Max  Lower  Upper | **573825**  509671  645108 | **1270019**  1051419  1614552 | **59983**  42668  77297 | **253327**  220246  291631 | **2007863**  1744135  2342280 | **108926**  79025  138827 | **738337**  570211  938873 |
|  | 2 | Max  Lower  Upper | **589334**  514306  659522 | **1291210**  1051604  1630047 | **61098**  44721  79994 | **258037**  220794  297053 | **2039653**  1776561  2341516 | **106600**  79044  142859 | **747937**  580812  952201 |
| 3 | 1 | Max  Lower  Upper | **421705**  373083  481548 | **917279**  775153  1109899 | **43316**  30662  56943 | **188417**  161011  218564 | **1746197**  1490930  2035126 | **83489**  62196  109264 | **644875**  522118  802706 |
|  | 2 | Max  Lower  Upper | **396962**  346582  445605 | **841698**  727040  1010212 | **41143**  29388  53802 | **176306**  152120  204312 | **1658641**  1395447  1906199 | **78599**  57778  100461 | **606472**  489473  747168 |
| 4 | 1 | Max  Lower  Upper | **362428**  319789  406772 | **855330**  724003  1039528 | **38616**  27076  51045 | **193084**  166840  221828 | **1825357**  1572084  2140030 | **82275**  62856  104760 | **480539**  378761  599765 |
|  | 2 | Max  Lower  Upper | **370903**  325735  412597 | **862545**  728777  1081439 | **39334**  27579  51994 | **197947**  168669  225952 | **1874928**  1611735  2143334 | **82763**  62983  104625 | **490954**  384321  612396 |
| 5 | 1 | Max  Lower  Upper | **551201**  471910  612588 | **1305745**  1101123  1579427 | **55250**  37943  73888 | **252081**  216471  293314 | **2376814**  2023841  2814194 | **134115**  103460  169369 | **587646**  450274  753364 |
|  | 2 | Max  Lower  Upper | **543211**  480683  621371 | **1314389**  1129410  1598370 | **56277**  38648  75262 | **256768**  220496  298767 | **2409278**  2077099  2807894 | **136609**  103823  169395 | **600792**  454203  762928 |
| 6 | 1 | Max  Lower  Upper | **458469**  405282  511656 | **1207337**  1000973  1481780 | **57028**  42633  73639 | **206554**  176935  239291 | **2375848**  2005669  2793895 | **102628**  79680  130676 | **557702**  418050  728186 |
|  | 2 | Max  Lower  Upper | **488946**  433982  553070 | **1295076**  1075222  1624857 | **61389**  45893  79269 | **225705**  192143  259267 | **2543776**  2179644  3041883 | **111848**  85773  143412 | **592537**  444159  781915 |
| 7 | 1 | Max  Lower  Upper | **620013**  546055  716158 | **1320146**  1051433  1901948 | **62234**  45552  81481 | **266447**  232125  306188 | **1710274**  1484702  1980220 | **98241**  68695  135174 | **625236**  446597  826993 |
|  | 2 | Max  Lower  Upper | **589012**  509699  677655 | **1286495**  1018232  1825353 | **60102**  43103  77100 | **252122**  217936  289726 | **1614825**  1397882  1877256 | **95755**  66399  126508 | **591622**  420598  786509 |
| 8 | 1 | Max  Lower  Upper | **595318**  519763  670872 | **1330021**  1046040  1733847 | **58989**  41360  77974 | **239586**  205223  277768 | **2049539**  1802104  2413239 | **97578**  67914  131925 | **616339**  458645  814012 |
|  | 2 | Max  Lower  Upper | **592712**  517158  670872 | **1283125**  1009565  1653081 | **58989**  41360  77974 | **239586**  203314  277768 | **2073388**  1787198  2431126 | **97578**  67914  131925 | **627445**  463088  825118 |
| 9 | 1 | Max  Lower  Upper | **401041**  352626  449547 | **885196**  738335  1072402 | **46620**  35700  59221 | **177973**  153140  205172 | **1630390**  1417362  1882150 | **77851**  59477  101062 | **390041**  296486  502857 |
|  | 2 | Max  Lower  Upper | **384215**  346190  438084 | **864286**  723275  1041738 | **45770**  34224  57315 | **173564**  151506  201427 | **1600632**  1396245  1840667 | **78329**  58391  99217 | **381571**  288373  484224 |
| 10 | 1 | Max  Lower  Upper | **528798**  462236  602756 | **1305355**  1061294  1685005 | **58384**  41703  76348 | **244770**  210448  286317 | **2242770**  1887772  2631048 | **108582**  80513  141083 | **631541**  473918  824891 |
|  | 2 | Max  Lower  Upper | **523987**  448959  586914 | **1245225**  1015300  1630047 | **56059**  40942  74955 | **240302**  208380  281092 | **2165531**  1853317  2597548 | **106600**  77593  137057 | **611761**  459079  791266 |
| 11 | 1 | Max  Lower  Upper | **621371**  540606  725584 | **1444655**  1197149  1765111 | **52209**  34580  71194 | **279677**  241495  321676 | **1814029**  1551688  2138974 | **106945**  74159  141293 | **640771**  469751  831781 |
|  | 2 | Max  Lower  Upper | **631792**  538000  725584 | **1402970**  1163280  1767716 | **52209**  34580  71194 | **281586**  241495  321676 | **1822972**  1557650  2118106 | **106945**  74159  142854 | **647434**  478635  834002 |
| 12 | 1 | Max  Lower  Upper | **582291**  498921  668267 | **1565803**  1065580  2670463 | **56277**  38648  76618 | **241495**  199496  283495 | **2025690**  1721613  2401315 | **110068**  80404  152221 | **674087**  429772  1038338 |
|  | 2 | Max  Lower  Upper | **585155**  513504  678037 | **1552453**  1165003  2603344 | **55942**  39367  76661 | **240152**  203205  284876 | **2032985**  1762730  2403447 | **113704**  83489  153461 | **700193**  421926  1091577 |

Table S3. The most likely demographic parameter value estimates (max) and the values of the limits on the associated 95% highest posterior density interval (lower, upper) for population migration rates (*2Nm*) from 24 IMa3 output files (12 datasets of ~200 loci each duplicated). The *2Nm* rates are understood as the rate by which genes of a population are supplanted by genes from another. For example, *2N_0_m_0>1_* is the rate at which the genes of *H. crassicornis* are supplanted by genes from *H. emurai*.

| Dataset | Duplicate | Estimate | *2N_0_m_0>1_* | *2N_0_m_0>2_* | *2N_1_m_1>0_* | *2N_1_m_1>2_* | *2N_1_m_1>3_* | *2N_2_m_2>0_* | *2N_2_m_2>1_* | *2N_3_m_3>1_* |
| --- | --- | --- | --- | --- | --- | --- | --- | --- | --- | --- |
| 1 | 1 | Max | 1.949E-01 | 4.266E-04 | 1.395E-05 | 1.514E-05 | 9.062E-02 | 1.382E-02 | 3.498E-05 | 7.720E-06 |
|  |  | Lower | 1.599E-01 | 0.000E+00 | 0.000E+00 | 0.000E+00 | 0.000E+00 | 1.749E-04 | 0.000E+00 | 0.000E+00 |
|  |  | Upper | 2.398E-01 | 1.814E-03 | 3.668E-03 | 4.375E-03 | 1.696E-01 | 4.453E-02 | 1.809E-02 | 1.875E-03 |
|  | 2 | Max | 2.010E-01 | 4.532E-04 | 1.335E-05 | 1.409E-05 | 8.533E-02 | 1.438E-02 | 3.498E-05 | 7.400E-06 |
|  |  | Lower | 1.633E-01 | 1.528E-05 | 0.000E+00 | 0.000E+00 | 0.000E+00 | 1.049E-04 | 0.000E+00 | 0.000E+00 |
|  |  | Upper | 2.441E-01 | 1.818E-03 | 3.699E-03 | 4.353E-03 | 1.756E-01 | 4.495E-02 | 1.872E-02 | 1.828E-03 |
| 2 | 1 | Max | 2.007E-01 | 3.342E-04 | 1.507E-05 | 1.500E-05 | 5.585E-02 | 3.498E-05 | 3.498E-05 | 8.100E-06 |
|  |  | Lower | 1.636E-01 | 0.000E+00 | 0.000E+00 | 0.000E+00 | 0.000E+00 | 0.000E+00 | 0.000E+00 | 0.000E+00 |
|  |  | Upper | 2.434E-01 | 1.581E-03 | 3.812E-03 | 3.734E-03 | 1.680E-01 | 2.011E-02 | 2.088E-02 | 1.985E-03 |
|  | 2 | Max | 2.031E-01 | 3.310E-04 | 1.657E-05 | 1.440E-05 | 5.576E-02 | 3.498E-05 | 3.498E-05 | 8.310E-06 |
|  |  | Lower | 1.667E-01 | 0.000E+00 | 0.000E+00 | 0.000E+00 | 0.000E+00 | 0.000E+00 | 0.000E+00 | 0.000E+00 |
|  |  | Upper | 2.449E-01 | 1.553E-03 | 4.027E-03 | 3.874E-03 | 1.673E-01 | 1.823E-02 | 1.935E-02 | 1.953E-03 |
| 3 | 1 | Max | 2.287E-01 | 3.085E-04 | 1.409E-05 | 1.269E-05 | 1.669E-04 | 9.340E-03 | 6.052E-03 | 8.420E-06 |
|  |  | Lower | 1.917E-01 | 0.000E+00 | 0.000E+00 | 0.000E+00 | 0.000E+00 | 0.000E+00 | 0.000E+00 | 0.000E+00 |
|  |  | Upper | 2.737E-01 | 1.492E-03 | 3.874E-03 | 3.565E-03 | 9.998E-02 | 3.880E-02 | 3.264E-02 | 2.264E-03 |
|  | 2 | Max | 2.299E-01 | 3.196E-04 | 1.384E-05 | 1.286E-05 | 1.769E-02 | 8.501E-03 | 7.031E-03 | 8.660E-06 |
|  |  | Lower | 1.905E-01 | 1.475E-05 | 0.000E+00 | 0.000E+00 | 0.000E+00 | 0.000E+00 | 0.000E+00 | 0.000E+00 |
|  |  | Upper | 2.769E-01 | 1.520E-03 | 3.585E-03 | 3.511E-03 | 8.463E-02 | 3.873E-02 | 3.390E-02 | 2.226E-03 |
| 4 | 1 | Max | 2.183E-01 | 4.596E-04 | 1.664E-05 | 1.437E-05 | 4.121E-02 | 3.498E-05 | 2.064E-03 | 8.630E-06 |
|  |  | Lower | 1.816E-01 | 1.622E-05 | 0.000E+00 | 0.000E+00 | 0.000E+00 | 0.000E+00 | 0.000E+00 | 0.000E+00 |
|  |  | Upper | 2.638E-01 | 1.844E-03 | 4.843E-03 | 4.296E-03 | 1.356E-01 | 3.131E-02 | 2.585E-02 | 2.183E-03 |
|  | 2 | Max | 2.176E-01 | 4.491E-04 | 1.969E-05 | 1.395E-05 | 5.516E-02 | 3.498E-05 | 3.253E-03 | 8.030E-06 |
|  |  | Lower | 1.797E-01 | 1.549E-05 | 0.000E+00 | 0.000E+00 | 0.000E+00 | 0.000E+00 | 0.000E+00 | 0.000E+00 |
|  |  | Upper | 2.600E-01 | 1.802E-03 | 4.863E-03 | 4.477E-03 | 1.433E-01 | 3.040E-02 | 2.704E-02 | 2.064E-03 |
| 5 | 1 | Max | 1.927E-01 | 9.459E-03 | 1.489E-05 | 1.388E-05 | 1.067E-02 | 3.498E-05 | 7.661E-03 | 9.120E-06 |
|  |  | Lower | 1.586E-01 | 3.536E-03 | 0.000E+00 | 0.000E+00 | 0.000E+00 | 0.000E+00 | 0.000E+00 | 0.000E+00 |
|  |  | Upper | 2.355E-01 | 1.860E-02 | 4.304E-03 | 4.233E-03 | 8.725E-02 | 2.165E-02 | 3.103E-02 | 2.453E-03 |
|  | 2 | Max | 1.946E-01 | 9.266E-03 | 1.433E-05 | 1.416E-05 | 6.206E-03 | 3.498E-05 | 8.151E-03 | 9.260E-06 |
|  |  | Lower | 1.582E-01 | 3.386E-03 | 0.000E+00 | 0.000E+00 | 0.000E+00 | 0.000E+00 | 0.000E+00 | 0.000E+00 |
|  |  | Upper | 2.347E-01 | 1.814E-02 | 4.572E-03 | 4.346E-03 | 8.530E-02 | 2.004E-02 | 3.103E-02 | 2.546E-03 |
| 6 | 1 | Max | 2.641E-01 | 3.964E-04 | 1.472E-05 | 1.262E-05 | 8.571E-02 | 3.498E-05 | 1.312E-02 | 7.750E-06 |
|  |  | Lower | 2.196E-01 | 0.000E+00 | 0.000E+00 | 0.000E+00 | 1.438E-02 | 0.000E+00 | 0.000E+00 | 0.000E+00 |
|  |  | Upper | 3.170E-01 | 1.825E-03 | 4.253E-03 | 3.546E-03 | 1.585E-01 | 4.341E-02 | 4.432E-02 | 2.070E-03 |
|  | 2 | Max | 2.668E-01 | 4.239E-04 | 1.402E-05 | 1.367E-05 | 7.712E-02 | 3.498E-05 | 1.781E-02 | 8.560E-06 |
|  |  | Lower | 2.204E-01 | 0.000E+00 | 0.000E+00 | 0.000E+00 | 1.330E-02 | 0.000E+00 | 0.000E+00 | 0.000E+00 |
|  |  | Upper | 3.188E-01 | 1.894E-03 | 3.995E-03 | 3.431E-03 | 1.585E-01 | 4.299E-02 | 4.600E-02 | 2.182E-03 |
| 7 | 1 | Max | 1.854E-01 | 6.783E-04 | 1.664E-05 | 1.962E-05 | 1.848E-01 | 7.346E-04 | 3.498E-05 | 7.610E-06 |
|  |  | Lower | 1.514E-01 | 5.402E-05 | 0.000E+00 | 0.000E+00 | 6.547E-02 | 0.000E+00 | 0.000E+00 | 0.000E+00 |
|  |  | Upper | 2.267E-01 | 2.143E-03 | 4.410E-03 | 6.297E-03 | 2.990E-01 | 1.956E-02 | 1.634E-02 | 1.865E-03 |
|  | 2 | Max | 1.889E-01 | 7.134E-04 | 1.598E-05 | 1.640E-05 | 1.870E-01 | 5.247E-04 | 3.498E-05 | 8.070E-05 |
|  |  | Lower | 1.530E-01 | 6.179E-05 | 0.000E+00 | 0.000E+00 | 7.271E-02 | 0.000E+00 | 0.000E+00 | 0.000E+00 |
|  |  | Upper | 2.280E-01 | 2.185E-03 | 4.298E-03 | 6.215E-03 | 3.039E-01 | 1.851E-02 | 1.627E-02 | 1.977E-03 |
| 8 | 1 | Max | 1.901E-01 | 1.428E-02 | 1.402E-05 | 1.321E-05 | 1.126E-01 | 3.498E-05 | 1.550E-02 | 8.490E-06 |
|  |  | Lower | 1.572E-01 | 6.851E-03 | 0.000E+00 | 0.000E+00 | 2.156E-02 | 0.000E+00 | 4.548E-04 | 0.000E+00 |
|  |  | Upper | 2.333E-01 | 2.508E-02 | 3.855E-03 | 3.871E-03 | 2.139E-01 | 3.026E-02 | 3.817E-02 | 1.757E-03 |
|  | 2 | Max | 1.922E-01 | 1.491E-02 | 1.521E-05 | 1.234E-05 | 1.088E-01 | 3.498E-05 | 1.529E-02 | 7.720E-06 |
|  |  | Lower | 1.555E-01 | 7.156E-03 | 0.000E+00 | 0.000E+00 | 3.862E-03 | 0.000E+00 | 0.000E+00 | 0.000E+00 |
|  |  | Upper | 2.340E-01 | 2.597E-02 | 3.878E-03 | 3.590E-03 | 1.974E-01 | 3.138E-02 | 3.838E-02 | 1.798E-03 |
| 9 | 1 | Max | 2.191E-01 | 6.227E-04 | 1.598E-05 | 1.356E-05 | 1.989E-02 | 2.123E-02 | 3.498E-05 | 9.500E-06 |
|  |  | Lower | 1.808E-01 | 3.421E-05 | 0.000E+00 | 0.000E+00 | 0.000E+00 | 4.023E-03 | 0.000E+00 | 0.000E+00 |
|  |  | Upper | 2.634E-01 | 2.415E-03 | 4.074E-03 | 3.838E-03 | 1.080E-01 | 5.363E-02 | 2.186E-02 | 2.480E-03 |
|  | 2 | Max | 2.206E-01 | 6.101E-04 | 1.577E-05 | 1.314E-05 | 2.141E-02 | 2.060E-02 | 3.498E-05 | 9.850E-06 |
|  |  | Lower | 1.820E-01 | 3.211E-05 | 0.000E+00 | 0.000E+00 | 0.000E+00 | 3.673E-03 | 0.000E+00 | 0.000E+00 |
|  |  | Upper | 2.649E-01 | 2.331E-03 | 3.895E-03 | 3.824E-03 | 9.998E-02 | 5.398E-02 | 2.305E-02 | 2.473E-03 |
| 10 | 1 | Max | 2.039E-01 | 7.168E-04 | 1.381E-05 | 1.293E-05 | 7.826E-02 | 1.291E-02 | 5.912E-03 | 7.860E-06 |
|  |  | Lower | 1.662E-01 | 8.741E-05 | 0.000E+00 | 0.000E+00 | 1.060E-03 | 0.000E+00 | 0.000E+00 | 0.000E+00 |
|  |  | Upper | 2.474E-01 | 2.174E-03 | 3.355E-03 | 3.712E-03 | 1.754E-01 | 4.432E-02 | 3.327E-02 | 1.941E-03 |
|  | 2 | Max | 2.048E-01 | 6.602E-04 | 1.405E-05 | 1.279E-05 | 7.186E-02 | 9.760E-03 | 3.498E-05 | 8.140E-06 |
|  |  | Lower | 1.681E-01 | 6.102E-05 | 0.000E+00 | 0.000E+00 | 0.000E+00 | 0.000E+00 | 0.000E+00 | 0.000E+00 |
|  |  | Upper | 2.472E-01 | 2.114E-03 | 3.387E-03 | 3.774E-03 | 1.626E-01 | 4.509E-02 | 3.740E-02 | 1.977E-03 |
| 11 | 1 | Max | 2.120E-01 | 9.381E-03 | 1.647E-05 | 1.304E-05 | 4.711E-02 | 2.134E-03 | 6.052E-03 | 8.070E-06 |
|  |  | Lower | 1.750E-01 | 3.776E-03 | 0.000E+00 | 0.000E+00 | 0.000E+00 | 0.000E+00 | 0.000E+00 | 0.000E+00 |
|  |  | Upper | 2.550E-01 | 1.722E-02 | 4.133E-03 | 3.898E-03 | 1.193E-01 | 3.117E-02 | 2.529E-02 | 1.896E-03 |
|  | 2 | Max | 2.100E-01 | 9.161E-03 | 1.454E-05 | 1.346E-05 | 3.684E-02 | 1.994E-03 | 7.731E-03 | 8.000E-06 |
|  |  | Lower | 1.740E-01 | 3.754E-03 | 0.000E+00 | 0.000E+00 | 0.000E+00 | 0.000E+00 | 0.000E+00 | 0.000E+00 |
|  |  | Upper | 2.566E-01 | 1.767E-02 | 4.057E-03 | 3.970E-03 | 1.113E-01 | 2.837E-02 | 2.606E-02 | 1.863E-03 |
| 12 | 1 | Max | 1.621E-01 | 2.514E-04 | 1.528E-05 | 1.594E-05 | 2.085E-01 | 3.498E-05 | 9.410E-03 | 9.360E-06 |
|  |  | Lower | 1.278E-01 | 0.000E+00 | 0.000E+00 | 0.000E+00 | 9.980E-02 | 0.000E+00 | 0.000E+00 | 0.000E+00 |
|  |  | Upper | 2.033E-01 | 1.304E-03 | 3.529E-03 | 4.575E-03 | 3.129E-01 | 2.627E-02 | 3.551E-02 | 2.050E-03 |
|  | 2 | Max | 1.629E-01 | 2.531E-04 | 1.440E-05 | 1.587E-05 | 2.058E-01 | 3.498E-05 | 8.291E-03 | 9.330E-06 |
|  |  | Lower | 1.277E-01 | 0.000E+00 | 0.000E+00 | 0.000E+00 | 1.105E-01 | 0.000E+00 | 0.000E+00 | 0.000E+00 |
|  |  | Upper | 2.046E-01 | 1.284E-03 | 3.557E-03 | 4.555E-03 | 3.166E-01 | 2.557E-02 | 3.600E-02 | 2.005E-03 |

Table S4. The most likely demographic parameter value estimates (max) and the values of the limits on the associated 95% highest posterior density interval (lower, upper) for divergence times (*t*) and effective population sizes (*N_e_*) from two IMa3 output files estimated from simulated ddRADSeq loci (two duplicate runs). The parameters simulated mimic the median empirical parameter estimates with population sizes, splitting times, and mutation rates scaled down 10 times. The estimate *t_0_* represents the most recent split in the northeastern Pacific to create *H. crassicornis* and *H. opalescens*; *t_1_* represents the most ancestral split to create the northeastern Pacific ancestor and *H. emurai*. The *N_e_* estimates numbered 0-4 represent *Hermissenda crassicornis*, *H. emurai*, *H. opalescens*, the northeastern Pacific ancestor, and the most recent common ancestor for all populations, respectively.

| Simulation | Duplicate | Estimate | *t_0_* | *t_1_* | *q_0_* | *q_1_* | *q_2_* | *q_3_* | *q_4_* |
| --- | --- | --- | --- | --- | --- | --- | --- | --- | --- |
| 10x scaled. | 1 | Max  Lower  Upper | **582627**  558653  609125 | **1965223**  1826428  2095607 | **64055**  56958  71862 | **312125**  291211  333494 | **2636596**  2494646  2789061 | **157483**  137895  178004 | **398880**  317496  500138 |
|  | 2 | Max  Lower  Upper | **583258**  559284  609125 | **1971532**  1826428  2101916 | **64055**  57312  71507 | **312125**  291211  333494 | **2636596**  2494646  2783804 | **156861**  137273  178004 | **398880**  315603  501085 |

Table S5. The most likely demographic parameter value estimates (max) and the values of the limits on the associated 95% highest posterior density interval (lower, upper) for population migration rates (*2Nm*) from two IMa3 output files estimated from simulated ddRADSeq loci (two duplicate runs). The parameters simulated mimic the median empirical parameter estimates with population sizes, splitting times, and mutation rates scaled down 10 times. The *2Nm* rates are understood as the rate by which genes of a population are supplanted by genes from another. For example, *2N_0_m_0_*_>1_ is the rate at which the genes of *H. crassicornis* are supplanted by genes from *H. emurai*.

| Simulation | Duplicate | Estimate | *2N_0_m_0>1_* | *2N_0_m_0>2_* | *2N_1_m_1>0_* | *2N_1_m_1>2_* | *2N_1_m_1>3_* | *2N_2_m_2>0_* | *2N_2_m_2>1_* | *2N_3_m_3>1_* |
| --- | --- | --- | --- | --- | --- | --- | --- | --- | --- | --- |
| 10x scaled | 1 | Max | 7.119E-3 | 1.367E-3 | 6.520E-4 | 1.671E-5 | 3.498E-5 | 7.793E-5 | 3.152E-5 | 1.412E-5 |
|  |  | Lower | 3.899E-3 | 9.663E-5 | 0.000E+00 | 0.000E+00 | 0.000E+00 | 0.000E+00 | 0.000E+00 | 0.000E+00 |
|  |  | Upper | 1.167E-2 | 5.591E-3 | 5.234E-3 | 4.095E-3 | 1.382E-2 | 2.736E-2 | 6.965E-3 | 4.364E-3 |
|  | 2 | Max | **7.105E-3** | **1.1353E-3** | **7.139E-4** | **1.706E-5** | **3.498E-5** | **7.706E-5** | **3.187E-5** | **1.419E-5** |
|  |  | Lower | 3.894E-3 | 9.971E-5 | 0.000E+00 | 0.000E+00 | 0.000E+00 | 0.000E+00 | 0.000E+00 | 0.000E+00 |
|  |  | Upper | 1.168E-2 | 5.655E-3 | 5.241E-3 | 4.214E-3 | 1.368E-2 | 2.735E-2 | 7.043E-3 | 4.357E-3 |


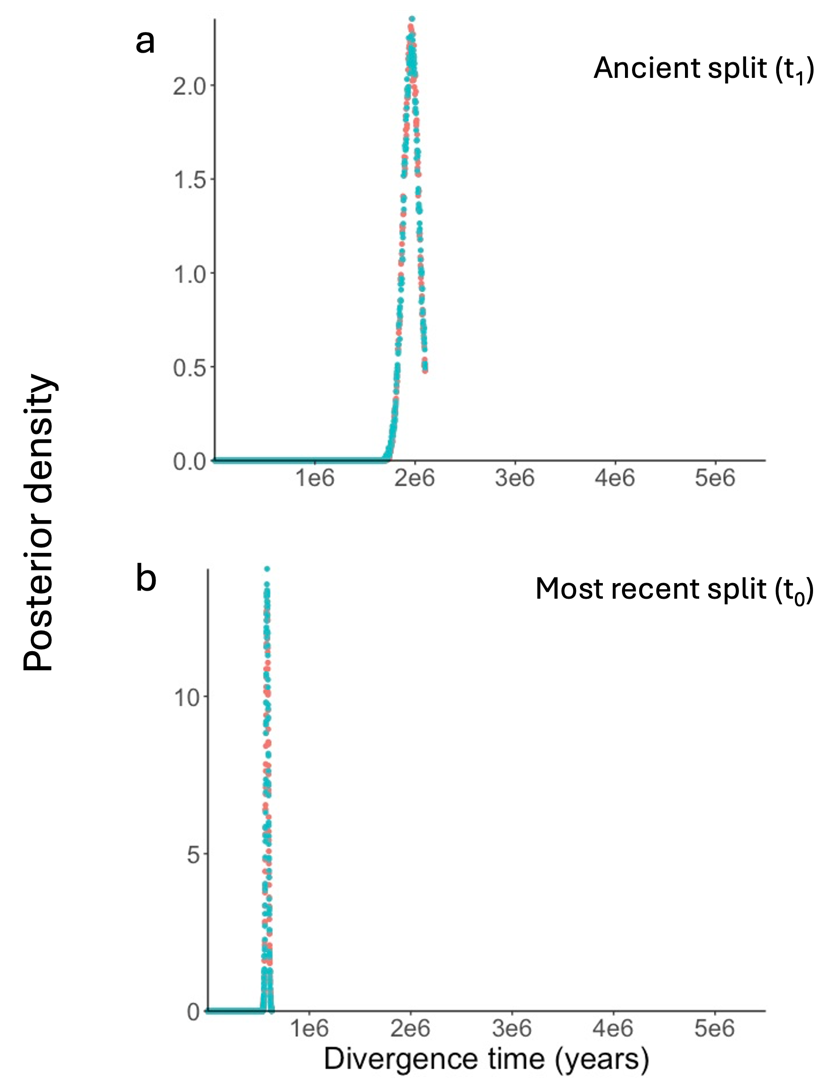


Figure S1. Posterior probability distributions estimated using IMa3 for the simulated dataset of Hermissenda nudibranchs. This simulation has demographic parameters scaled 10 times lower than the empirical estimated parameters. Figure 1a shows the more ancient split (set to 1.29 mya in simulations) in the genus and 1b the more recent split (set to 0.55 mya in simulations). Colour indicates 1 of 2 independent model runs with the simulated 200-locus dataset.


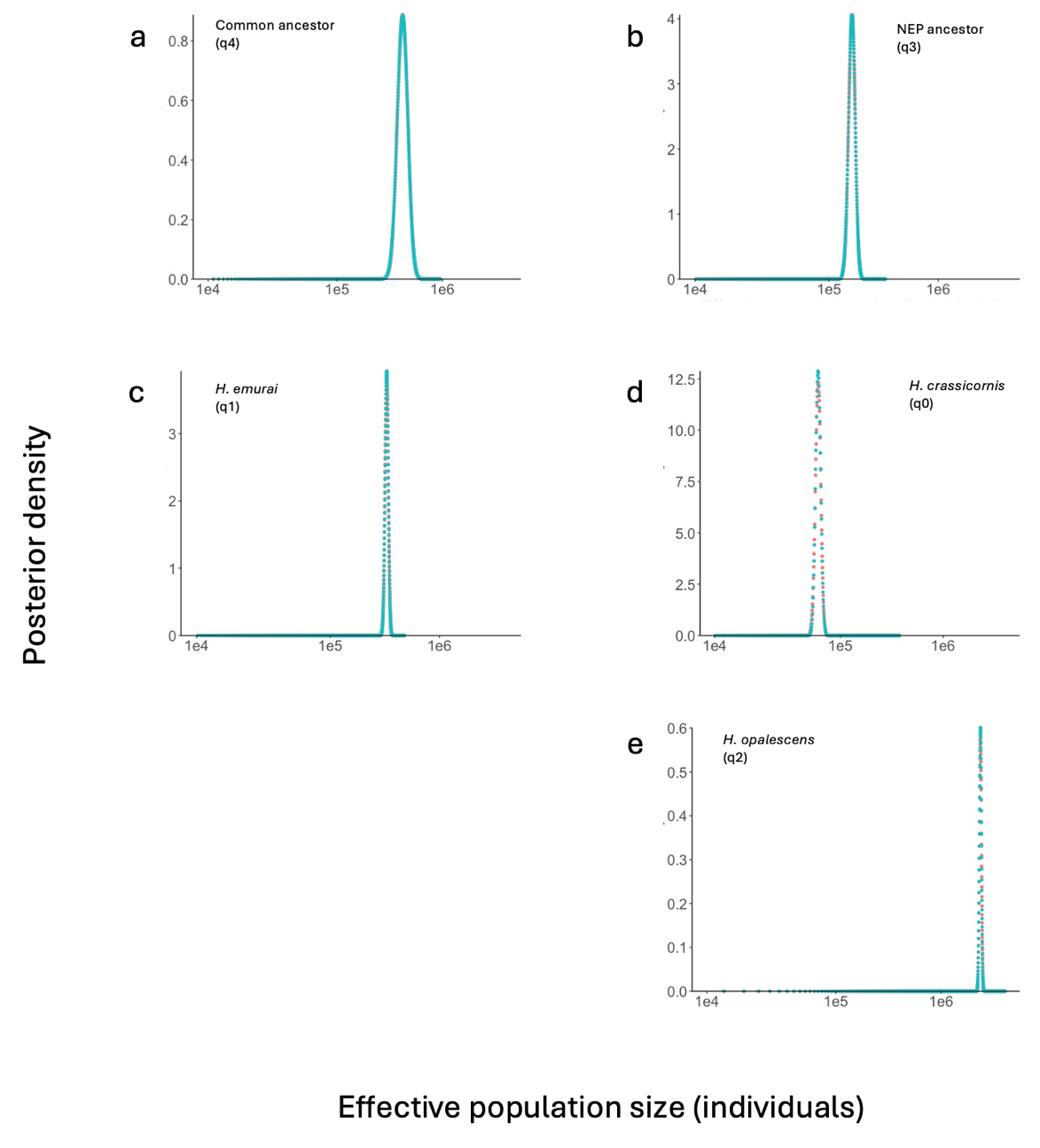


Figure S2. Posterior probability distributions estimated using IMa3 for the effective population sizes (individuals) for Hermissenda nudibranchs using the 10 times scaled down simulated dataset. Colour indicates 1 of 2 independent model runs with the simulated 200-locus dataset.


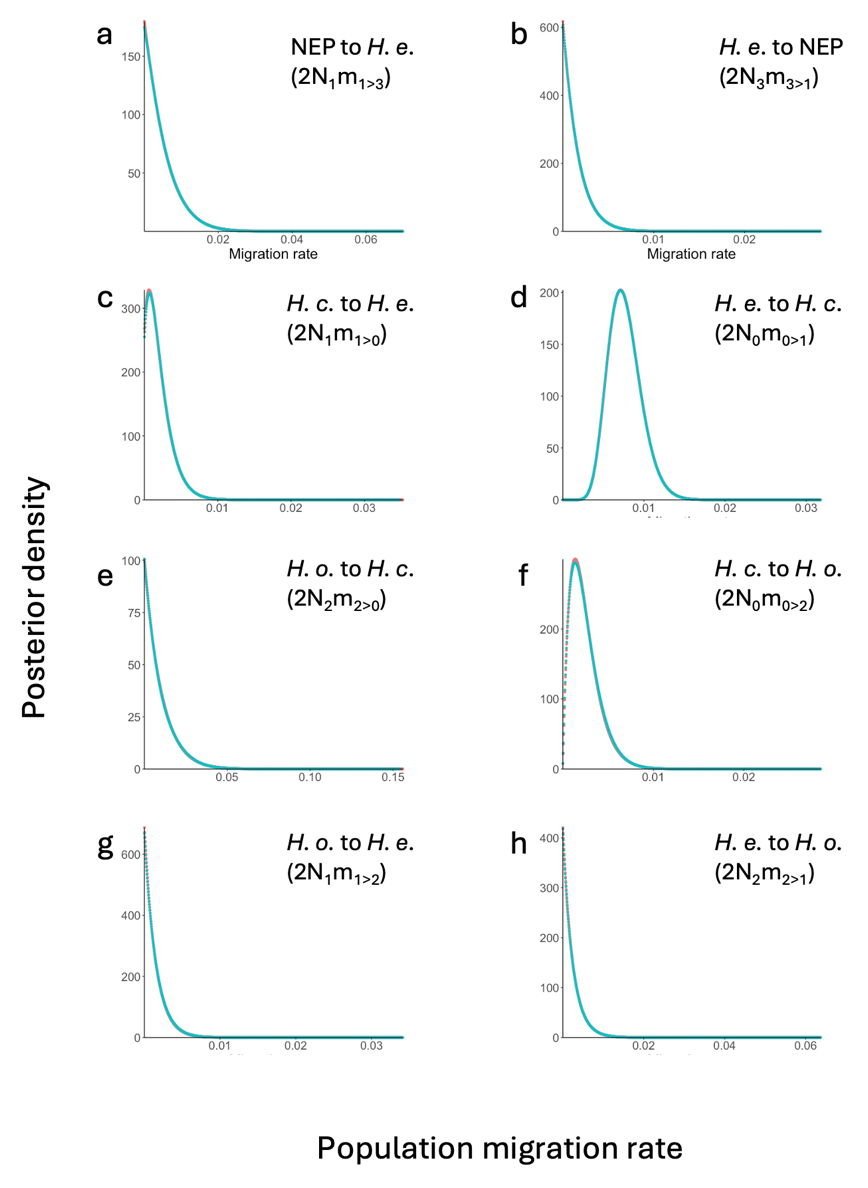


Figure S3. Posterior probability distribution estimated using IMa3 for the population migration rates the 10 times scaled simulated dataset of 200 loci representing Hermissenda nudibranchs. Colour indicates 1 of 2 independent model runs.
